# A Log File Analysis on the Validity of Partial Solutions in Figural Matrices Tests

**DOI:** 10.3390/jintelligence11020037

**Published:** 2023-02-16

**Authors:** Dominik Weber, Florian Krieger, Frank M. Spinath, Samuel Greiff, Johanna Hissbach, Nicolas Becker

**Affiliations:** 1 Department of Individual Differences & Psychodiagnostics, Saarland University, Campus A1 3, D-66123 Saarbrücken, Germany; 2 Department of Methods of Educational Research, TU Dortmund University, Emil-Figge-Straße 50, D-44227 Dortmund, Germany; 3 Department of Behavioral and Cognitive Sciences, University of Luxembourg, 4366 Luxembourg, Luxembourg; 4 Department of Biochemistry and Molecular Cell Biology, University Medical Centre Hamburg-Eppendorf, Martinistraße 52, D-20246 Hamburg, Germany; 5 Department of Individual Differences & Psychodiagnostics, Greifswald University, Franz-Mehring-Str. 57, D-17489 Greifswald, Germany

**Keywords:** intelligence, figural matrices, partial solutions, computer-based testing, construction-based tests, log files, sequence analysis, grade point average, validation

## Abstract

As a component of many intelligence test batteries, figural matrices tests are an effective way to assess reasoning, which is considered a core ability of intelligence. Traditionally, the sum of correct items is used as a performance indicator (total solution procedure). However, recent advances in the development of computer-based figural matrices tests allow additional indicators to be considered for scoring. In two studies, we focused on the added value of a partial solution procedure employing log file analyses from a computer-based figural matrices test. In the first study (*n* = 198), we explored the internal validity of this procedure by applying both an exploratory bottom-up approach (using sequence analyses) and a complementary top-down approach (using rule jumps, an indicator taken from relevant studies). Both approaches confirmed that higher scores in the partial solution procedure were associated with higher structuredness in participants’ response behavior. In the second study (*n* = 169), we examined the external validity by correlating the partial solution procedure in addition to the total solution procedure with a Grade Point Average (GPA) criterion. The partial solution procedure showed an advantage over the total solution procedure in predicting GPA, especially at lower ability levels. The implications of the results and their applicability to other tests are discussed.

## 1. Introduction

### 1.1. Reasoning as a Key Component of General Intelligence

Intelligence is an important predictor of a broad set of life domains, such as school grades (Roth et al. 2015) or job performance (Schmidt and Hunter 2004). After a long history of investigating the structure of intelligence, since the beginning of the millennium, there has been a broad scientific consensus. Due to great similarities between the longstanding predominant assumptions of the Cattell–Horn *g_f_-g_c_ theory* (Cattell 1963; Horn 1968) and Carroll’s *three stratum theory* (Carroll 1993), they have been consolidated under the umbrella term *Cattell–Horn–Carroll theory of cognitive abilities* (*CHC theory*; McGrew 2009). The CHC theory assumes a hierarchically structured intelligence model with a global factor of general intelligence (*g*). According to the positive manifold outlined by Spearman (Spearman 1904), g is composed of 16 broad abilities that are intercorrelated but distinct in content. The broad abilities differ in their factor loadings on the higher-ordered g, i.e., in their contributions to general intelligence. Important broad abilities are comprehension–knowledge, short-term memory and visual processing (McGrew 2009). However, the largest contribution to g typically provides reasoning ability (e.g., Carroll 1993; Cattell 1963; Keith 1990), which is employed to solve novel problems that cannot be solved on using acquired knowledge (McGrew 2009). In CHC theory, the broad abilities are further separated into several narrower abilities, such as induction or quantitative reasoning within reasoning. Given the high factor loading, reasoning is considered not an exhaustive but at least a valid proxy for general intelligence.

### 1.2. Assessment of Reasoning through Matrices Tests

**Figure 1:**
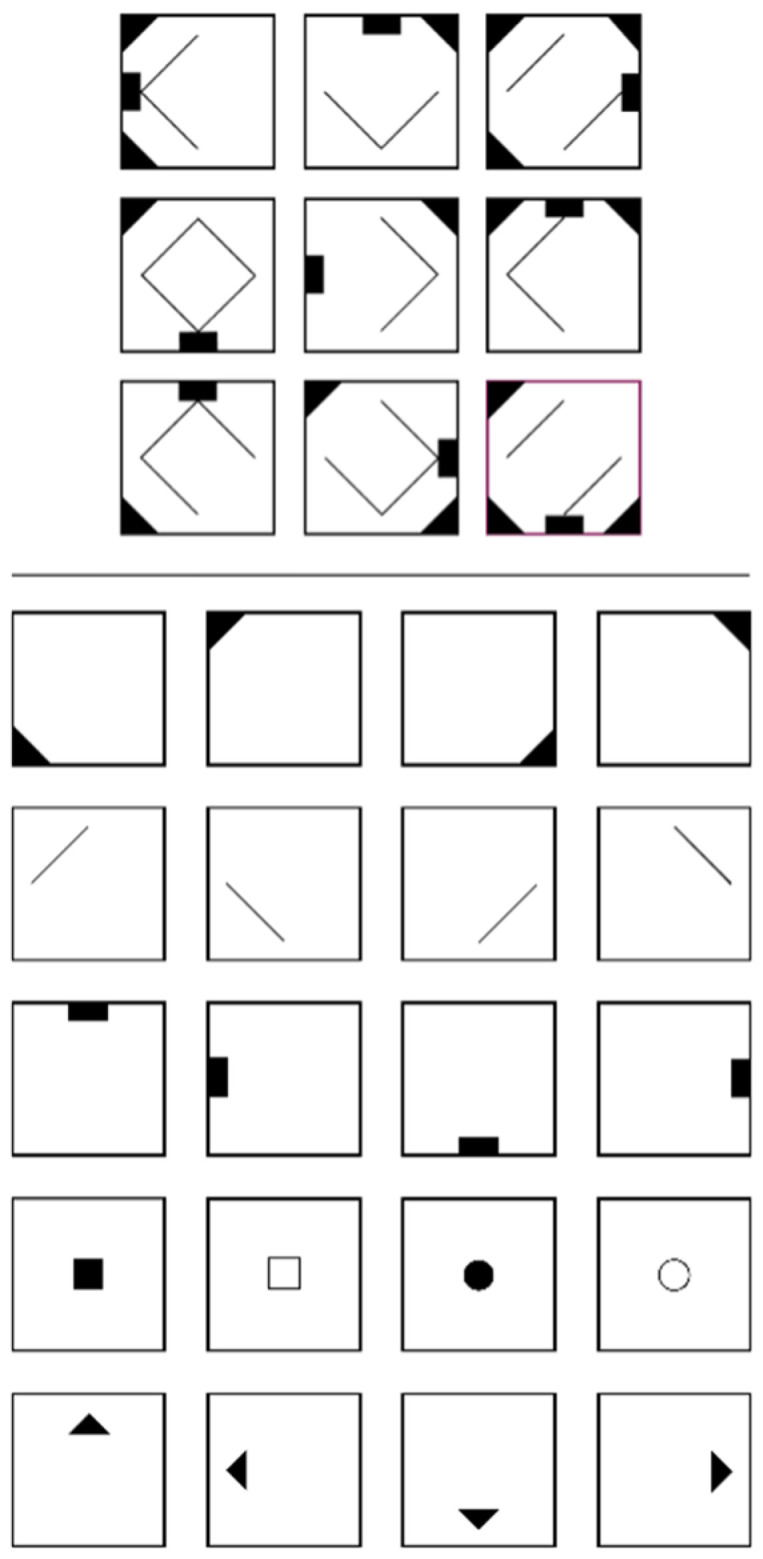
The figure shows an item with three rules (solution field in violet filled for demonstration purpose): (rule 1) The triangles in the corners of the first and second cells sum up in the third cell in each row. (rule 2) The rectangle rotates around the edges of the cells in each row. (rule 3) Only lines that are present in the first or the second cell are present in the third cell in each row.

Preferably, reasoning is operationalized with figural matrices items (e.g., Jensen 1998; Marshalek et al. 1983) which are content of many psychometric intelligence tests (e.g., Beauducel et al. 2010; Cattell 1949; Formann et al. 2011; Raven and Raven 2003; Wechsler 2003, 2008). Figural matrices items commonly consist of a 3 × 3 matrix filled with geometrical symbols that follow certain rules (e.g., symbols in the first two cells of a row are summed in the third cell, see Figure 1). The last cell is usually left empty, and respondents must indicate the symbols that logically complete the matrix based on the induced rule(s).

Snow (1980) proposed two principal solution strategies that individuals may use in figural reasoning tasks, which in the meantime have been well documented and proven (e.g., Bethell-Fox et al. 1984; Hayes et al. 2011): constructive matching and response elimination. In constructive matching, participants engage in a top-down strategy in which they try to comprehend the logical rules contained in the items and based on this, mentally construct the solution on their own. Response elimination, by contrast, is considered a bottom-up strategy, in which participants one by one eliminate the response options with respect to the item stem based on inappropriate distractors and choose one of the remaining ones.

There is broad evidence that constructive matching is associated with higher scores on matrices tests and results in better validity and g-saturation of test performance than response elimination (e.g., Arendasy and Sommer 2013; Vigneau et al. 2006). Therefore, especially over the last 15 years, there have been efforts to develop construction-based matrices items that prevent participants from using response elimination strategies. Unlike distractor-based items, construction-based ones are not provided with response options for resolution. For instance, as part of their research, Mitchum and Kelley (2010) developed a paradigm in which participants were asked to draw the contents of the last cell of the matrix by themselves. More recent advances provide a construction kit with all possible matrices symbols, which is supposed to be employed by the participants to compose the solution on their own (Becker and Spinath 2014; Krieger et al. 2022). Recently, the so-called Open Matrices Item Bank (Koch et al. 2022) became available, which offers a non-profit set of 220 construction-based matrices items for research and application purposes. One advantage of these construction-based approaches, in addition to the enhancement of validity, is the substantial reduction in the probability to solve the items by random.

### 1.3. Partial Scoring in Matrices Tests

More importantly, there is a chance to use construction-based responses to obtain more detailed information about the participants’ processing behavior, which opens up new possibilities for scoring. When evaluating performance in figural matrices tests, traditionally, points are awarded only if all rules are solved correctly (total solution procedure). If at least one of the rules employed in the items is not solved correctly, no points are awarded. In other words, if an item contains five rules, respondents who correctly solve four rules, one rule, or no rules would receive the same total score for this item (i.e., 0). In turn, it is reasonable to consider a procedure that accounts for solutions to single rules (partial solution procedure), which may lead to a better differentiation of figural matrices test performance. For instance, Figure 1 shows a three-rule item (rule 1: addition; rule 2: rotation; rule 3: disjunctive union) with three corresponding symbol groups (group 1: corners; group 2: rectangles; group 3: lines). Using the total solution procedure, the required symbols of all three rules must be selected in order to obtain a point. By contrast, using the partial solution procedure, participants already receive a point if the required symbols of a single rule (rule 1, 2 or 3) have been selected.

Consequently, Research Question 1 (RQ1) asked whether applying the partial solution procedure offers a substantial gain in information on items with multiple rules compared with the total solution procedure.

### 1.4. Structuredness in Item Processing

Relatedly, we asked whether this potential improvement in differentiation by applying the partial solution procedure is meaningful for the diagnostic process. Previous studies have indicated interindividual differences in test-taking behavior on figural matrices tests and their influence on test outcomes. In fact, Carpenter et al. (1990) found that respondents with higher intellectual ability were capable of solving more rules on items with multiple rules. In extensive research using think-aloud protocols, eye-tracking methods and computer simulations they have identified two key sub-processes in processing matrices when participants use constructive matching strategies: one of them is rule induction, as described above. However, the ability for rule induction alone is not sufficient to clarify why individuals perform differently on items with multiple rules, since items with only one rule often elicit ceiling effects (i.e., almost all participants solve the item), whereas the item difficulties increase with the number of rules (e.g., Embretson 1998; Primi 2001). According to Carpenter et al. (1990), in addition to rule induction, the second sub-process for items with multiple rules is goal management. It is assumed that in order to successfully solve an item (global goal), it is necessary to separate it into sub-goals. Regarding matrices tests, solving a single rule is considered as a sub-goal. Once the rules have been solved, they must be maintained in working memory until the solutions of all rules contained in an item are achieved.

Although it has been shown that the crucial component in goal management is not the maintenance of the *solutions* of the sub-goals (Domnick et al. 2017; see also Unsworth and Engle 2005; Wiley et al. 2011) successful item processing seems to be associated with the ability to systematically *separate* the item into sub-goals and *process* these sub-goals (Krieger et al. 2019; Loesche et al. 2015). Evidence for the role of this structured process was also found by Hayes et al. (2011), who used eye-tracking methods to shed more light on participants’ processes during item processing. They were able to uncover strong correlations of two behavioral measures with a performance on the RAPM (Raven and Raven 2003): One of them was the extent of toggling during item processing. Toggling was negatively correlated with the RAPM score, indicating that higher-performing participants engaged more persistently with a particular rule. The other measure was termed the systematic component of item processing. Results suggested that serial processing of the items was associated with a higher RAPM score.

Beyond the separation of the global target into sub-goals, there is evidence that selective encoding also plays a role in processing items with multiple rules (Meo et al. 2007; Primi 2001). Serving as a cognitive filtering function, selective encoding refers to the challenge of focusing on (currently) relevant item information and neglecting (currently) irrelevant information. As a consequence, participants with a higher selective encoding ability remain more persistent with a rule without being distracted by the other rules, which results in a more structured solution process (Krieger et al. 2019; Meo et al. 2007; Primi 2001). Thus, a structured behavior in the sense of adequate goal management and the ability of selective encoding seems to be critical for successful item processing.

Therefore, Research Question 2 (RQ2) asked whether the ability to solve more partial solutions is associated with more structured behavior. An affirmative answer would provide internal validity of the partial solution procedure for discriminating figural matrices test performance.

### 1.5. External Perspective on Partial Solutions

In addition to the internal validation that is particularly relevant for the diagnostic process, we investigated whether the partial solution procedure has practical relevance. For this purpose, we examined the associations between the partial solutions and an intelligence-related external criterion in comparison to the total solutions. In this regard, we focused on Grade Point Average (GPA), which shows a robust correlation with intelligence (Roth et al. 2015). In particular, performance in matrices tests (i.e., as one proxy for intelligence) is considered a relevant predictor of GPA (e.g., Downey et al. 2014; Gralewski and Karwowski 2012; Mõttus et al. 2012; Roth et al. 2015). Therefore, Research Question 3 (RQ3) asked whether the partial solution procedure showed any practical advantage over the total solution procedure in predicting GPA. Affirmative results would add practical value to the partial solution procedure with potential implications for (1) test administration and (2) further research on log files for the diagnostic process in general.

### 1.6. Research Goals

The goals of this research were to determine (1) whether partial solutions can differentiate between participants beyond total solutions in the context of intelligence testing (RQ1) and (2) whether this differentiation is meaningful in terms of both internal and external validity (RQ2 and RQ3). To this end, participants completed computer-based versions of a construction-based matrices test as a well-established proxy for reasoning, which has critical importance for general intelligence. This matrices test allowed partial solutions to be computed for each item. We conducted two studies: In Study 1, we examined the internal validity of the partial solutions by assessing participants’ processing behavior using log files and evaluating it by means of sequence analyses (thus covering RQ1 and RQ2). In Study 2, we further examined the external validity of the partial solutions by investigating their potential to predict Grade Point Average (GPA) beyond traditional scores based on total solutions (thus covering RQ3).

## 2. Study 1

In Study 1, we addressed RQ1 and RQ2 in order to examine the internal validity of the partial solution procedure. To answer both research questions, we analyzed log files from computer-based figural matrices tests. For RQ1, we investigated whether the partial solution procedure could provide additional differentiation compared with the total solution procedure. Therefore, we examined the variability of the partial scores at different levels of the total scores. We expected the partial scores to scatter at identical total scores and thus to provide additional differentiation of performance.

For RQ2, we first applied an exploratory bottom-up approach and investigated whether there were qualitative differences in test-taking behavior in terms of structuredness, i.e., in the homogeneity of the rule sequences during item processing (RQ2a). For this, we employed sequence analyses. We expected to find indicators of more structuredness when more rules are solved. The findings were consolidated with a confirmatory top-down approach: By means of log files, we examined how often the participants switched between the rules contained in the matrices (*rule jumps*) while processing (RQ2b). The literature suggests that individuals with higher cognitive abilities are better at splitting a task into subgoals through adequate goal management (e.g., Carpenter et al. 1990; Hayes et al. 2011; Loesche et al. 2015) and at processing these subgoals sequentially without being distracted by currently irrelevant information (Krieger et al. 2019; Meo et al. 2007; Primi 2001). Thus, we expected to find strong negative correlations between rule jumps and the partial sum score, indicating a benefit of engaging in more structured behavior.

### 2.1. Method

#### 2.1.1. Sample and Test Procedure

We assessed *n* = 198 university students (149 female, 49 male) on individual computers as part of a larger cognitive screening and provided monetary compensation. Participants were on average *M* = 23.00 (*SD* = 4.43) years old. Prior to the actual test, participants had to complete a practice item. Subsequent to this item they were allowed to start the test. Subsequently, all participants worked on the items of the computer-based Design a Matrices test (DESIGMA; Becker and Spinath 2014). A feature of this test is that it has a distractor-free response format. This means that participants cannot select their solution from a set of alternatives but must construct it themselves as described above. For this purpose, they can choose from six symbol groups with four symbols each (e.g., symbol group: squares; symbols: square in the upper left, upper right, lower left or lower right corner). Each symbol group is associated with a particular inference rule per item (e.g., addition, subtraction, intersection). We included 18 representative items in our analyses. Of the 18 items, ten involved two rules, five involved three rules, two involved four rules and one involved five rules. Once a symbol is clicked, it appears in the solution field. If clicked a second time, it disappears. Clicking RESET deletes all symbols from the solution field. Each step in the construction process was tracked and stored in a log file. Consequently, we could pinpoint when each participant behaviorally tried to solve a certain rule.

#### 2.1.2. Statistical Analysis

For each of the 18 items, both the total solution procedure and the partial solution procedure were applied. To address RQ1, results from both procedures were summed across the items. More importantly, we performed a White test for heteroscedasticity with the partial sum score regressed on the total sum score. This was done to determine whether the variance in the total partial score varied as a function of the level of the total sum score. This would indicate that the partial score could additionally differentiate between participants in a certain ability range based on their total sum score.

**Figure 2:**
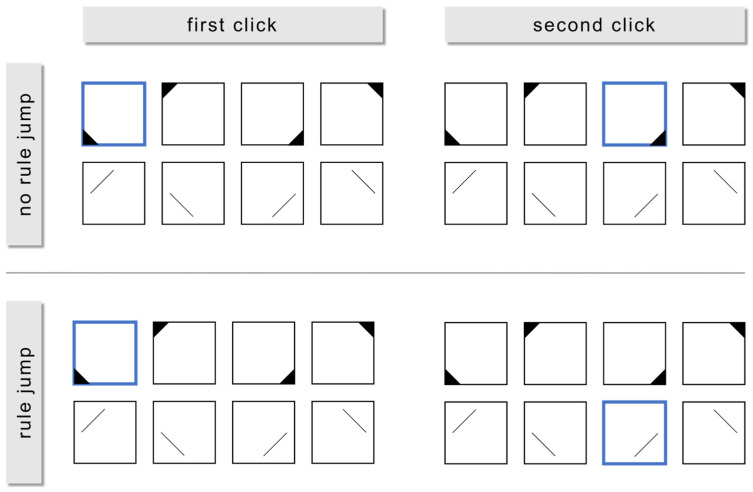
The upper section of the figure shows an example of a click (framed in blue) sequence without any rule jump, since the second click selects a symbol of the same symbol group as the first click. By contrast, the lower section shows an example of a click sequence with a rule jump, since the second click selects a symbol of a different symbol group than the first click.

To address RQ2a, the total sample for each item was divided according to the number of rules solved correctly. We performed a sequence analysis to qualitatively analyze processing behavior using the *TraMineR* (Gabadinho et al. 2011) package in *R* (R Core Team 2021). Sequence analyses are a method for visualizing and organizing log file data. They display sequences of events of interest (e.g., clicks) to enable a judgement on the structuredness of the events by inspecting these visualizations. We chose this method as an exploratory precursor to quantitative analysis of structuredness in order to provide a qualitative impression of structuredness. All participants’ solution sequences were visualized. By dividing up the sample according to the number of rules they solved, it was possible to identify potential differences in structuredness between respondents who solved different numbers of rules. To address RQ2b, the number of rule jumps (i.e., how often a participant switched their responses between rules) was computed by extracting the sequence of symbols clicked in the item construction kit from the log files. For each item and participant, the log files were an array in which the sequence of individual clicks on the construction kit was stored (e.g., *participant_15_item_7: [group_1_symbol_1, group_1_symbol_2, group_1_symbol_4, etc.]*). We chose this approach to record behavioral item processing in a naturalistic way. Each time the participants clicked on a symbol that belonged to a certain rule and subsequently clicked on a symbol that belonged to another rule, this was considered a rule jump (Figure 2). Then, we correlated this rule jump score with the partial sum score.

### 2.2. Results

#### 2.2.1. Descriptive Statistics and Internal Consistencies

Table 1 presents descriptive statistics for the 18 items in terms of total and partial scores. Item difficulties ranged from *p_min_* = .13 to *p_max_* = .61. Internal consistencies were α = .89 (95% CI [.87, .91]) for the total solution procedure and α = .92 (95% CI [.91, .94]) for the partial solution procedure. The item–rest correlations of all items in both procedures were above *r_i(t−i)_* = .30.

#### 2.2.2. Relationship between Total and Partial Sum Scores (RQ1)

**Figure 3:**
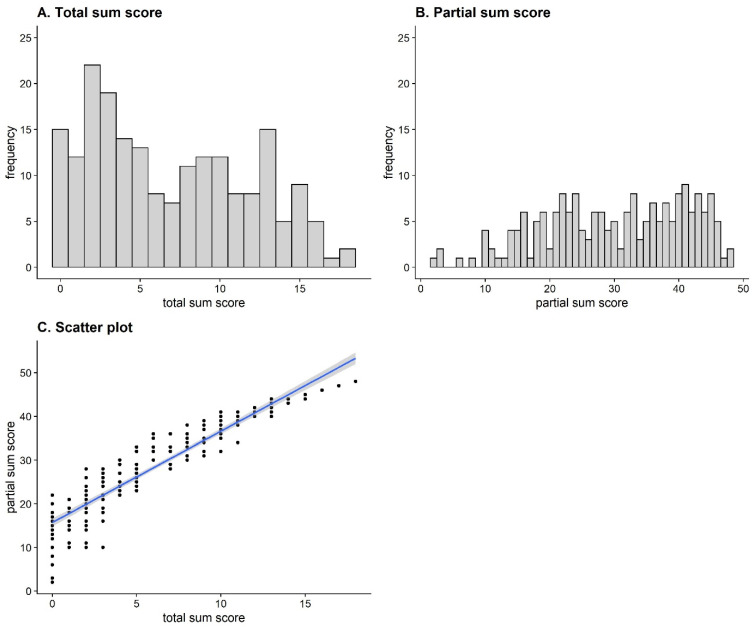
Histogram of total sum scores (**A**), histogram of partial sum scores (**B**) and scatterplot for the relationship between the total and partial sum scores (**C**).

Figure 3A shows the frequency distribution of total sum scores for all participants. On average, the participants solved *M* = 6.89 (*SD* = 4.94) items. The distribution reveals a skewness of *g* = .35 and an excess of γ = −1.09. Thus, it is slightly right skewed and approximately in line with the distribution of the DESIGMA norm sample (*g_desigma_* = .33, γ_desigma_ = −1.37; Becker and Spinath 2014). To compare the range of the sample in this study with the range of the norm sample, we calculated a range ratio to control for the different number of items by dividing the empirical range by the number of points that could be achieved. For both samples, this yielded *RR* = 1.00, implying that the entire range of values was covered, and no range restriction was present.

Figure 3B shows the frequency distribution for the partial sum score. On average, participants solved *M* = 30.12 (*SD* = 11.01) rules correctly. Figure 3C shows the relationship between the total and partial sum scores (*r* = .94, 95% CI [.92, .95], *p* < .001). Despite a strong correlation, the figure illustrates that the partial sum scores were more scattered for participants with fewer correct total solutions and became more homogeneous as the number of correct total solutions increased. The White test for homoscedasticity provided inferential statistical support for this effect, *BP*(2) = 25.02, *p* < .001. Thus, heteroscedasticity could be assumed.

#### 2.2.3. Sequence Analyses (RQ2a)

**Figure 4:**
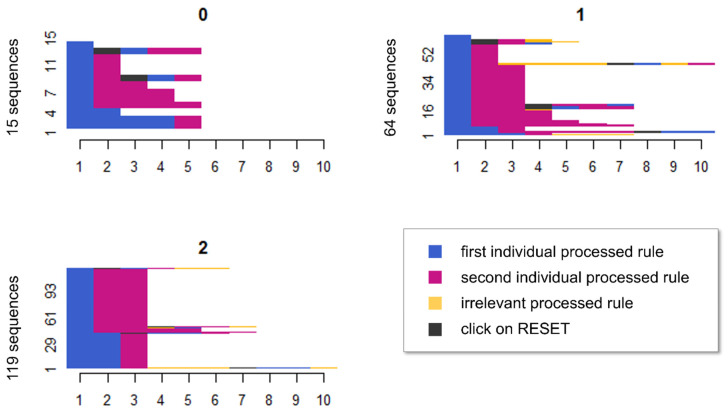
The figure shows the sequence diagrams for each number of correct partial solutions. The number to the left of the diagram represents the number of participants with the respective number of correctly solved partial solutions (in the example: 15 participants did not achieve any partial solution, 64 participants achieved one partial solution and 119 participants achieved two partial solutions). To provide a clearer structure, the participants’ sequences are ordered in such a way that identical sequences are placed next to each other; *x*-axis = number of clicks, *y*-axis = number of participants, blue = first individual processed rule, red = second individual processed rule, yellow = third individual processed rule; gray = click on RESET.

To obtain a visual overview of participants’ processing behavior, we computed a sequence analysis for each item. The sequence analyses revealed different processing patterns depending on the number of rules solved. A visual inspection revealed that the more systematically participants worked on an item, the more rules they solved. Figure 4 illustrates this by showcasing item 10. This item contained two rules, and thus, participants were divided up according to whether they solved no rules, one rule, or both rules correctly. The *x*-axis shows the number of clicks, and the *y*-axis the number of participants. The blue area indicates a click on a symbol of the first individual processed rule, the red area refers to the second individual rule. The yellow area refers to an irrelevant symbol group (i.e., a symbol group that was not associated with a relevant rule in this item). Gray represents a click on RESET within a sequence. In the diagram for zero correctly solved rules, the 15 participants largely selected correct symbol groups but ultimately did not or only partially select the correct symbols of these symbol groups. This can be inferred from the observation that the sequences in the diagram predominantly consist of the two required symbol groups of this two-rule item (blue and red sections). Since the participants achieved a partial score of zero, they apparently clicked on the wrong, on too few or on too many symbols within these symbol groups. Within some sequences, participants addressed earlier rules after another rule had already been processed. This can be observed by the fact that in some sequences single symbol groups were not clicked sequentially but unstructured (e.g., the sequence *blue > red > black > blue > red*). Thus, these participants jumped between rules. Sixty-four participants were able to solve one of the two rules. In this subgroup, participants also mainly dealt with the correct symbol groups, but in one symbol group, they selected the wrong symbols. A slightly smaller proportion in this group jumped between rules. By contrast, of the 119 participants who solved both rules correctly, almost all processed the item systematically; only a small proportion jumped between different rules. The sequence analyses for the other items are provided on the OSF (https://osf.io/r6h7v/, accessed on 15 February 2023).

#### 2.2.4. Rule Jumps (RQ2b)

Finally, the correlation between rule jumps and the number of correctly solved rules was tested for significance. Across the 18 items we examined, the deviation from the required number of rule jumps was significantly negatively related to the number of correct partial solutions with a strong effect, *r* = −.54, 95% CI [−.44, −.64], *p* < .001. Thus, the more systematically participants processed an item, the more rules they solved correctly.

### 2.3. Discussion

The aim of Study 1 was to examine whether the partial solution procedure is internally valid. To this end, we investigated whether partial solutions on items in the context of intelligence testing could discriminate between participants above and beyond total solutions. In addition, we aimed to determine whether this improved differentiation was meaningful for individual item processing.

Exploratory and confirmatory analyses revealed that the partial solution procedure went beyond the total solution procedure in differentiating between participants especially at lower ability levels. In addition, sequence analyses indicated that participants with more systematic processing behavior achieved more partial solutions. The strong negative correlation between rule jumps and the number of partial solutions provided a second piece of evidence in support of this assumption.

These findings are in line with commonly reported evidence on processing behavior on inductive reasoning items: Carpenter et al. (1990) proposed besides the actual rule induction goal management as an important sub-process in item processing. Repeatedly, it has been demonstrated that the ability to separate a matrices item (global goal) into its contained rules (sub-goals) and to process them sequentially is associated with higher cognitive ability (e.g., Bethell-Fox et al. 1984; Hayes et al. 2011; Loesche et al. 2015). This goes along with the observation that selective encoding ability, which prevents (currently) irrelevant information from distracting from processing the (currently) relevant sub-goal (i.e., rule), is critical for successful item processing (Krieger et al. 2019; Meo et al. 2007; Primi 2001). When considering only the total score, parts of the information about the solution process are neglected, because regardless of the number of achieved sub-goals (i.e., solved rules) 0 points are awarded if at least one of several rules was not solved. In contrast, the partial solution procedure seems to reflect the information about the participants’ ability for goal management and selective encoding more closely, which can be deduced from the strong correlation to the rule jumps as a measure of structuredness.

## 3. Study 2

Study 1 provided evidence for the internal validity of the partial solution procedure. The aim of Study 2 was to answer RQ3 by investigating the external validity of the procedure in addition to the internal validity just demonstrated. To this end, we focused on GPA as a well-established intelligence-related external criterion (Roth et al. 2015) and examined whether partial solutions are associated with GPA more strongly than total solutions. Based on the results from Study 1, we had three hypotheses:

**Hypothesis** **1.** *Total solutions and partial solutions are highly correlated, but also have substantial unshared variance. This was already reflected in the results of Study 1. The total scores and partial scores are computed from the same data and are thus naturally linked. Nevertheless, Study 1 already showed an unshared variance component of the two scores of 12.11%. Therefore, we did not assume multicollinearity despite a strong correlation (H1a). Furthermore, the White test in Study 1 indicated heteroscedasticity of the correlation between the two scores. At lower ability levels, the partial scores scattered much more widely than at upper ability levels, and also differentiated between participants with identical total scores. To corroborate these findings, in Study 2, we assumed that this effect would replicate and that there would also be heteroscedasticity in the correlation between the two scores, with a broader scatter of the partial scores at lower ability levels (H1b).*

**Hypothesis** **2.** *Based on the entire sample from Study 2, there is a significant advantage of the partial solution procedure over the total solution procedure in predicting GPA. We deduced this hypothesis from the observation in Study 1 that partial solutions were very strongly related to the structuredness of item processing, thus providing one strong indicator of internal validity. Specifically, we hypothesized the correlation between GPA and the partial solutions with GPA would be significantly higher than the correlation with the total solutions.*

**Hypothesis** **3.** *There are variations in the advantage of the partial solution procedure depending on participants’ ability level. Based on the findings on heteroscedasticity in Study 1, we expected the correlation of partial solutions with GPA to exceed the correlation of total solutions with GPA specifically at lower ability levels (H3a). At higher ability levels, we assumed no significant advantage of the partial solution procedure over the total solution procedure (H3b).*

### 3.1. Method

#### 3.1.1. Sample and Test Procedure

The sample for Study 2 consisted of *n* = 169 participants (153 female, 12 male, 4 non-binary) who were on average *M* = 19.62 (*SD* = 3.00) years old. The assessment was conducted in the context of training for the German medical student selection procedure (Studierendenauswahl-Verbund 2021) and was administered on individual computers. Participants were required to correctly solve two practice items before the beginning of the test. Then, participants were presented with 28 items from one version of the DESIGMA (Becker and Spinath 2014). Three of the items contained one rule, six items contained two rules, ten items contained three rules, six items contained four rules and three items contained five rules. After taking the test, participants received individual feedback about their performance.

After the training, participants were asked to report their GPA. Since all participants had requested to study at a German university, GPA refers to the German equivalent of the final high school diploma, which serves as a university entrance certificate. It is calculated as a weighted average of the grades in 40 courses in the ultimate two years of high school and a final exam (Kultusministerkonferenz 2022). In our study, 141 of the 169 participants received their certificate at a general high school and 28 at a different school (e.g., high school with a specific major). It was awarded in all 16 German federal states (Baden-Wuerttemberg: 29; Bavaria: 22; Berlin: 5; Brandenburg: 4, Bremen: 1; Hamburg: 6; Hesse: 12; Mecklenburg-Western Pomerania: 2; Lower Saxony: 8; North Rhine-Westphalia: 46; Rhineland-Palatinate: 13; Saarland: 2; Saxony: 4; Saxony-Anhalt: 2; Schleswig-Holstein: 3; Thuringia: 4; not reported: 6).

#### 3.1.2. Statistical Analysis

In this study, we addressed RQ3 and investigated whether partial solutions are associated with GPA more strongly than total solutions and whether this potential advantage is differentially meaningful at different ability levels. Therefore, we first calculated both a total sum score and a partial sum score for each participant. Based on Study 1, we assumed that the two scores were highly correlated but not multicollinear (H1a). For this purpose, we calculated the variance inflation factor (VIF), which should be less than the critical threshold of 10 (e.g., Marquardt 1970; Neter et al. 1989). To corroborate the results on heteroscedasticity from Study 1, we performed the White test by regressing the partial scores on the total scores. This was to confirm the observation that partial scores differentiate between participants with identical total scores, especially at lower ability levels (H1b).

In Study 1, a higher partial score was strongly correlated with a more structured item processing. Based on this finding, we hypothesized that the partial solution procedure has an external advantage over the total solution procedure (H2). To this end, we computed the correlations of the two scores with GPA and conducted a *t*-test for paired correlations to determine whether the partial scores correlate significantly higher with GPA than the total scores (please note that due to the negative polarity of grades in the German school system, lower GPA values indicate better performance). The *t*-test for paired correlations takes into account not only the two bivariate correlations (*partial scores × GPA* and *total scores × GPA*) but also the shared variance of two dependent variables (*partial scores × total scores*). This allowed us to examine inferentially whether the partial scores share unique variance with GPA over and above the total scores (Revelle 2021).

Based on the findings on heteroscedasticity indicating that partial scores achieve higher discrimination, especially at the lower ability level, we assumed that the partial scores have practical relevance here in particular (H3). To examine this hypothesis, we first divided the sample according to the total scores into a subsample of *n_A_* = 81 (47.93%) with lower scores and a subsample of *n_B_* = 88 (52.07%) with higher scores (please note that an exact median split was not possible due to tied ranks resulting from identical scores among some participants). Using this strategy, we adapted the approach by Stadler et al. (2020) by restricting the ability variance and examining whether additional log files (in our case the partial solution procedure) accounted for additional variance in an external criterion such as GPA. In detail, to test our hypothesis that the partial solution procedure has an external advantage at lower ability levels (H3a); in particular, we compared the correlations of the total solution procedure and partial solution procedure with GPA by conducting a *t*-test for paired correlations on the lower ability sub-sample. To test our hypothesis that neither procedure has an advantage at higher ability levels (H3b), we conducted the same analysis for the higher ability sub-sample.

### 3.2. Results

#### 3.2.1. Descriptive Statistics and Internal Consistencies

Table 2 shows the descriptive statistics of the 28 items from Study 2, separated into total scores and partial scores. Item difficulties ranged from *p_min_* = .22 to *p_max_* = .89. Internal consistencies were α = .92 (95% CI [.91, .94]) for the total solution procedure and α = .95 (95% CI [.94, .96]) for the partial solution procedure. The item–rest correlations of all items apart from items 1, 2 and 7 (total solution procedure), respectively, items 1, 2 and 6 (partial solution procedure) were above *r_i(t-i)_* = .30. On average, the participants solved *M* = 17.08 (*SD* = 7.48) of the 28 items correctly in the total solution procedure and *M* = 57.01 (*SD* = 22.02) of 84 rules in the partial solution procedure. Participants had a mean GPA of *M* = 1.67 with a standard deviation of *SD* = 0.48.

The distribution of the total solution procedure had a skewness of *g* = −.52 and an excess of γ = −0.98. Therefore, in this study, the distribution was slightly left skewed and not slightly right skewed as in the DESIGMA norm sample (*g_desigma_* = .33; Becker and Spinath 2014), while the excess of both samples is in alignment (γ_desigma_ = −1.37). As within Study 1, to compare the range of the sample in this study with the range of the norm sample, we calculated a range ratio correcting for different numbers of items by dividing the empirical range by the maximum number of points that could be achieved. For the sample in this study this was *RR* = .93 and for the DESIGMA norm sample *RR* = 1.00. Thus, both ranges were very high and similar, so that no range restriction was assumed.

#### 3.2.2. Relationship between Total and Partial Sum Scores

The total scores and partial scores were highly correlated (*r* = .94, *p* < .001), but the variance inflation factor at *VIF* = 8.48 was below the critical threshold of 10. Hence, in accordance with H1a, we concluded that there was no multicollinearity between the two procedures. The White test for homoscedasticity was significant, *BP*(2) = 32.34, *p* < .001. Thus, in accordance with H1b, heteroscedasticity was found.

#### 3.2.3. Practical Value of the Partial Solution Procedure Compared to the Total Solution Procedure

In accordance with H2, the partial scores correlated significantly more strongly with GPA (*r* = −.18) than the total scores (*r* = −.13), *t*(166) = 1.91, *p* = .029. Since we had an a priori directional hypothesis, we report the one-sided *p*-value here. Thus, we found evidence for H2. In H3, we expected that the external advantage of the partial solution procedure would be particularly noticeable at lower ability levels. In accordance with hypothesis H3a, in the lower ability subsample, partial scores were associated significantly higher with GPA (*r* = −.16) than total scores (*r* = −.07), *t*(78) = 1.69, *p* = .048. We report the one-sided *p*-value here as well, since we had an a priori directional hypothesis. On the other hand, as assumed in H3b, in the higher ability subsample, there was no significant difference in the correlations between total scores (*r* = .00) versus partial scores (*r* = −.08) and GPA, *t*(85) = 1.05, *p* = .299. Consequently, also H3 was considered confirmed.

### 3.3. Discussion

The aim of Study 2 was to provide evidence for the external advantage of the partial solution procedure compared to the total solution procedure. To this end, we focused on GPA as an established intelligence-related external criterion (e.g., Roth et al. 2015).

We investigated the practical advantage of partial scores compared to total scores in predicting GPA and whether this advantage is especially relevant for the lower ability range.

Based on the total sample, it was shown that partial solutions show significantly higher correlations with GPA. After separating the sample into more and less able participants, we found that the practical advantage of the partial solution procedure is particularly important for the lower ability range. This result is even more remarkable because the partial scores and total scores are calculated from identical data. Although there is no multicollinearity, the two scores are closely related. The fact that the partial scores still have an external advantage in predicting GPA, indicates the added value of the partial solution procedure compared to the total solution procedure for certain relevant outcomes.

It should be mentioned that the correlation between figural reasoning and GPA found in this study is somewhat lower than in other studies in which matrices tests were employed (cf. Downey et al. 2014: r = .46; Gralewski and Karwowski 2012: r = .15; Mõttus et al. 2012: r = .43). One possible cause for this might be that the participants received their high school diploma in different German federal states. Although measures such as the standardization of final exams have been taken in the German school system since 2005 to increase comparability (Kultusministerkonferenz 2009), there are still certain differences (e.g., different degrees of freedom in the weighting of courses) across the federal states. The fact that a significant effect was still found in our study, despite this unsystematic error variance, supports the advantage of the partial solution procedure. Nevertheless, future research could use larger samples to examine, how much the advantage of the partial solution procedure might benefit from systematizing this error variance, e.g., by means of higher linear modeling methods.

## 4. General Discussion

### 4.1. Consolidation of the Studies

The goal of this research was to introduce the partial solution procedure as a complementary approach to evaluating intelligence test items and to examine its internal and external validity. For this purpose, the partial solution procedure was compared to the traditional total solution procedure in two studies using an established figural matrices test (Becker and Spinath 2014; Krieger et al. 2022). In contrast to the total solution procedure, which only distinguishes dichotomously between solved and not solved items, the partial solution procedure differentiates gradually between different numbers of partial solutions within each item.

In Study 1, which addressed internal validity, heteroscedasticity was confirmed, with the partial solutions scattering more broadly among participants with identical total solutions, especially at lower ability levels. To address the question of whether this additional variance has relevance in terms of internal validity, it jumps between various rules within matrices were considered as a measure of structuredness. This revealed that the partial solution procedure was very strongly associated with the structuredness of item processing. Therefore, in Study 1, the internal validity of the partial solution procedure was inferred.

In Study 2, which addressed external validity, the finding that the partial solutions exhibited larger variance at lower ability levels was replicated. To answer the question of whether this larger variance is also practically meaningful, GPA was considered a well-established intelligence-related external criterion. Across the entire ability range of the sample, partial solutions correlated more strongly than total solutions with GPA. When separating the participants by ability level, the practical advantage of the partial solution procedure was shown to be particularly important at lower ability levels. Consequently, in Study 2, the external validity of the partial solution procedure was inferred.

### 4.2. Limitations and Future Prospects

It should first be mentioned that both samples were not assessed in high-stakes settings. Therefore, biasing variables such as performance motivation or concentration could have influenced the results (see Bates and Gignac 2022). However, in Study 2, we attempted to homogenize personal motivation by ensuring that participants had an intrinsic interest in participating due to the test setting (i.e., training for a student selection procedure) and that they received individual feedback on their performance after the assessment.

Furthermore, how the item processing instructions were designed in the two studies should be considered. The instructions each contained practice items. Only after these items had been solved completely, were participants allowed to start with the actual test. If anything, this indicated that total solutions and not partial solutions were the desired performance criterion. We chose this form of instruction because we were interested in a naturalistic solution process. However, this might stress the advantage of the partial solution procedure even more. If the participants believed that only the total solution matters, they would probably resign if they cannot solve a particular partial solution. On the other hand, if participants know that the partial solutions are also relevant, this might reduce resignation and rigidity effects, so that the responses reflect intelligence in a less biased way. Thus, future studies could follow up on this and point out the importance of instructing participants to seek out partial solutions.

Moreover, although the literature has not (yet) revealed different processing behavior for different inductive rules they are associated with varying difficulties (Carpenter et al. 1990; Preckel 2003; Vodegel Matzen et al. 1994). The goal of our research was the initial introduction of a novel procedure for scoring and its internal and external validation on a global level. It would be interesting if subsequent research focused on the internal aspects and particularly investigated whether certain processing patterns are applied to different rules.

In addition, how the solution process was measured should be noted. We used log files to record when a participant clicked on certain symbols. It would also be interesting, within the scope of future research, to assess additional behavioral indicators of problem-solving strategies, such as think-aloud prompts and eye movement data.

Finally, the item format should be considered. A major advantage of distractor-free items is that when solutions are generated using a construction kit, the probability of making a correct guess is reduced. In DESIGMA, the probability of generating the correct answer by clicking on the construction kit at random is *p* < .001. If points are awarded for partial solutions for single symbol groups, the guessing probability increases to *p* = .063 (due to 16 possible combinations of four symbols within a symbol group). However, compared to the common item format in which several distractors are provided (e.g., *p* = .200 with four distractors and one correct solution), the guessing probability is also vastly lower in the partial solution procedure. Nevertheless, future research may investigate whether the advantage of the partial solution procedure varies depending on the number of symbols and thus the guessing probability.

Along with this, it should be mentioned that all symbols of a symbol group were displayed in the same row. By doing so, we followed the common response format of validated construction-based matrices tests (Becker and Spinath 2014; Koch et al. 2022; Krieger et al. 2022). In addition, Levacher et al. (2021) were able to show that the construct validity of matrices tests is not compromised regardless of whether or not participants receive instruction on the rules contained in the items and their implications for the symbol groups prior to the assessment. Nevertheless, it would be an interesting prospect to examine whether, for instance, a randomized order of the symbols in the construction kit might have an impact on the results.

### 4.3. Applicability of the Partial Solution Procedure to Other Intelligence Components

Recent advances in test development employing construction-based item responding enable partial scores to be computed. The main goal of this research was to examine the validity of this partial solution procedure for matrices tests, as they are a frequently used operationalization of reasoning (e.g., Jensen 1998; Marshalek et al. 1983), which plays an important role in general intelligence (e.g., Carroll 1993; Keith 1990). In two studies, we found substantial initial evidence. Future research might aim to extend the results on the validity of the partial solution procedure to other intelligence components and their assessment methods. For this purpose, we would like to provide three suggestions considering the established CHC theory (McGrew 2009), moving from a proximal to a distal transfer: (1) first on a further operationalization of inductive reasoning; (2) second on a test of another narrow ability of reasoning; (3) third on a test of another broad ability of the CHC theory.

(1) Number sequences: Aside from matrices tests, number sequences are an alternative operationalization of inductive reasoning as a narrow ability of reasoning in CHC theory and are part of various intelligence tests (e.g., *LPS-2*, Kreuzpointner et al. 2013; *IST*, Beauducel et al. 2010). They consist of a sequence of numbers that usually follow several logical rules. The task of the participants is to detect those numbers within the sequence that violate the rules. An example of this would be the number sequence *31 4 29 6 27 8 35 10 22* (Item 16 of subtest 4 of the LPS-2, Kreuzpointner et al. 2013). The sequence contains two rules that refer alternately to each second number: The first rule requires subtracting 2 from the number to the next but one (31 − 2 = 29, etc.) and the second rule requires adding 2 from the number to the next but one (4 + 2 = 6, etc.). The last number in the sequence (22) violates the first rule by being 3 less than its reference number (25) rather than 2. Points are traditionally awarded for these items, as well as for matrices items, only if the total solution has been achieved, regardless of how many of the rules have been correctly recognized. A partial solution procedure could be applied by adapting the habitual approach of the Attention Swiping Task (Koch et al. 2021). This would require participants to assign numbers to different rules by marking (pen-and-paper version) or swiping (computer-based version) and labeling the rules. This would allow partial scores to be computed, which, in contrast to the total solution procedure, could provide more detailed information about inductive reasoning ability.

(2) Tests on quantitative reasoning: In addition to inductive reasoning, quantitative reasoning is a narrow ability of reasoning in CHC theory. It refers to the ability to reason based on numbers, mathematical operators and relations (cf. Carroll 1993). Tests of quantitative reasoning are found in various intelligence tests (e.g., *KABC-II*, Lichtenberger and Kaufman 2010; *WAIS-IV—Figure Weight*, Wechsler 2008; *WISC-IV—Figure Weight*, Wechsler 2003). An example of a quantitative reasoning item would be: “A craftsman gets paid EUR 60,- during the first 3 h of work. For each additional hour, he demands EUR 75,-. How many hours will he have to work, in order to earn EUR 255,-?” (Arendasy et al. 2006). The task requires participants to recognize several aspects of the item (requested quantitative variable, several partial equations, composing the partial equations) in order to solve the item. However, participants usually receive a point only if they determine the correct final result, and not if they have solved at least some sub-problems correctly. By tracking the sub-processes of these tasks it would be possible to apply a partial solution procedure to obtain more differentiated information about quantitative reasoning ability.

(3) Tests on visual processing: Visual processing is, besides reasoning, one of the broad abilities in CHC theory, which is preferably operationalized by items that require participants to perceive and transform figural and/or geometric stimuli (cf. McGrew 2009). For instance, there are so-called mental rotation tests (e.g., Shepard and Metzler 1971; Vandenberg and Kuse 1978), which display a two-dimensional projection of a three-dimensional cube figure in the item stem. In addition, different response options are offered, of which the item stem is to be found by mentally rotating the cube figure around the three spatial axes at several angular degrees. Just et al. (2001) revealed in an in-depth fMRI study that goal management functions are involved in mental rotation and that brain activity varies in strength and localization depending on the axis of rotation. Expanding on traditional distractor-based mental rotation tests (Shepard and Metzler 1971; Vandenberg and Kuse 1978), Thissen et al. (2020) introduced a construction-based approach that showed advantages over the conventional response format in terms of construct validity. Following on from this and considering the research of Just et al. (2001), mental rotation tasks as an operationalization of visual processing might be transformed into a format to which the partial solution procedure can be applied: Participants might be shown an unrotated primary configuration in which the single cubes of the cube figure are assigned different symbols. Next to it, a final configuration of the same cube figure, rotated in three axes, might be displayed, but containing only one of the symbols displayed in the primary configuration (serving as an anchor). The task of the participants might now be to fill the remaining cubes of the rotated final configuration with the correct symbols using a construction kit. For this purpose, participants might be offered the possibility to display up to two blank cube figures without symbols, which were rotated in only one axis as an intermediary processing step and which they could fill up by means of the construction kit. By doing so, the sub-goals arising from the goal management might be manifested, which might enable a more detailed test performance to be acquired by the partial solution procedure.

## 5. Conclusions

In summary, this research provides evidence for both the internal and external validity of the partial solution procedure. It may prove beneficial to consider partial solutions in a variety of research and application contexts, particularly when more fine-grained differentiation in the lower ability range is necessary. Potential application fields might be besides educational issues cognitive syndromes such as *Mild Cognitive Impairment* (*MCI*). MCI refers to debilitating cognitive alterations that are greater than expected for the given individual’s age and pose a high risk of transition to dementia (Gauthier et al. 2006; Petersen 2011). Therefore, it is of crucial importance to provide sensitive diagnostics for the early detection and progression of MCI. One approach may be intelligence diagnostics, since it has been shown that MCI patients at a certain stage of disease already show poorer performance, for instance, on matrices tests than healthy individuals (e.g., Borella et al. 2017; Chang et al. 2021; Chao et al. 2020; Chow et al. 2022; Jefferson et al. 2008). Thus, in order to facilitate more detailed information about cognitive abilities in the context of MCI, the partial solution procedure might be able to contribute, which would be an interesting scope of future research. In addition, the partial solution procedure could be advantageous in application fields, in which it is desirable to assess systematic test-taking behavior (e.g., specific contexts in personnel selection).

Finally, based on the results, we recommend that researchers consider the partial solution procedure in addition to the total solution procedure in both test construction and administration. Due to the advancing computerization of intelligence assessment with the possibility of automated test evaluation, it has become quite simple to apply the partial solution procedure without any actual additional effort. Embedding items in a format that enables partial scoring will allow for further evaluations of the partial solution procedure (e.g., Becker and Spinath 2014; Koch et al. 2022; Krieger et al. 2022). Subsequent research could examine the influence of high-stakes settings as well as differences in test instruction and item format. Further, there are many potential approaches to transfer the partial solution procedure to other tests.

## Figures and Tables

**Table 1:** Descriptive Statistics and Internal Consistencies.

Item	NoR	Total Solutions			Partial Solutions			
		*p*	α_dropped_	*r_i(t−i)_*	*M*	*SD*	α	*r_i(t−i)_*
1	2	.54	.89	.31	1.49	0.59	.92	.37
2	2	.57	.89	.44	1.50	0.63	.92	.46
3	2	.53	.89	.52	1.44	0.70	.92	.62
4	2	.33	.89	.63	1.16	0.69	.92	.66
5	2	.30	.88	.41	1.19	0.62	.92	.44
6	2	.39	.89	.47	1.20	0.73	.92	.54
7	2	.51	.89	.49	1.26	0.83	.92	.56
8	2	.13	.89	.47	0.91	0.59	.92	.48
9	2	.15	.89	.49	0.69	0.72	.92	.54
10	2	.59	.89	.64	1.53	0.63	.92	.64
11	3	.32	.88	.51	2.03	0.88	.92	.58
12	3	.46	.89	.58	2.22	0.88	.92	.63
13	3	.43	.88	.62	2.21	0.87	.92	.69
14	3	.35	.88	.65	1.85	0.99	.92	.74
15	3	.61	.88	.63	2.16	1.17	.92	.75
16	4	.21	.89	.50	2.23	1.31	.91	.81
17	4	.26	.88	.66	2.22	1.38	.91	.81
18	5	.21	.89	.51	2.83	1.61	.92	.79

Note: The sample size was *n* = 198 for all items; NoR = Number of Rules, *p* = item difficulty, α_dropped_ = Cronbach’s α when item was dropped, *r_i(t−i)_* = item–rest correlation, *M* = mean score, *SD* = standard deviation.

**Table 2:** Descriptive Statistics and Internal Consistencies.

Item	NoR	Total Solutions			Partial Solutions			
		*p*	α_dropped_	*r_i(t−i)_*	*M*	*SD*	α	*r_i(t−i)_*
1	1	.82	.93	.21	0.82	0.38	.95	.18
2	1	.89	.93	.29	0.89	0.31	.95	.21
3	1	.42	.92	.45	0.42	0.50	.95	.38
4	2	.75	.92	.44	1.72	0.51	.95	.38
5	2	.64	.93	.35	1.54	0.67	.95	.34
6	2	.56	.92	.42	0.91	0.29	.95	.28
7	2	.89	.93	.28	1.86	0.43	.95	.37
8	2	.72	.92	.44	1.68	0.57	.95	.49
9	2	.74	.92	.55	1.64	0.65	.95	.58
10	3	.49	.92	.52	2.12	1.04	.95	.61
11	3	.53	.92	.54	2.13	1.11	.95	.54
12	3	.64	.92	.64	2.39	0.98	.95	.73
13	3	.70	.92	.54	2.45	0.98	.95	.67
14	3	.75	.92	.58	2.52	0.96	.95	.70
15	3	.73	.92	.65	2.49	0.97	.95	.74
16	3	.50	.92	.63	2.12	1.05	.95	.74
17	3	.76	.92	.63	2.53	0.95	.95	.74
18	3	.59	.92	.58	2.20	1.14	.95	.75
19	3	.62	.92	.70	2.17	1.20	.94	.83
20	4	.49	.92	.47	2.11	1.10	.95	.77
21	4	.46	.92	.63	2.71	1.55	.94	.84
22	4	.64	.92	.63	2.24	1.24	.94	.81
23	4	.56	.92	.56	2.74	1.70	.94	.79
24	4	.43	.92	.60	2.46	1.71	.94	.84
25	4	.59	.92	.67	1.95	1.36	.94	.82
26	5	.22	.92	.46	2.71	2.06	.94	.82
27	5	.45	.92	.69	2.76	2.34	.95	.78
28	5	.47	.92	.60	2.70	2.39	.94	.70

Note: The sample size was *n* = 169 for all items; NoR = Number of Rules, *p* = item difficulty, α_dropped_ = Cronbach’s α when item was dropped, *r_i(t−i)_* = item–rest correlation, *M* = mean score, *SD* = standard deviation.

## Data Availability

Publicly available datasets were analyzed in this study. This data can be found here: https://osf.io/r6h7v/, accessed on 15 February 2023.
